# Resolution of Hashimoto thyroiditis with Janus kinase inhibitor therapy in a patient with alopecia universalis

**DOI:** 10.1210/jcemcr/luag074

**Published:** 2026-04-24

**Authors:** Susanne Ursula Trost, Angela Radulescu

**Affiliations:** Division of Diabetes, Endocrinology and Metabolism, University of Minnesota, Minneapolis, MN 55455, USA; Division of Diabetes, Endocrinology and Metabolism, University of Minnesota, Minneapolis, MN 55455, USA

**Keywords:** autoimmunity, thyroid peroxidase antibodies (TPO antibodies), Hashimoto thyroiditis, hypothyroidism, Janus kinase (JAK) inhibitors

## Abstract

Hashimoto thyroiditis (HT) is the most common autoimmune thyroid disorder, marked by lymphocytic inflammation and progressive hypothyroidism. Its pathophysiology involves both T-cell-mediated tissue destruction and the production of autoantibodies against thyroid peroxidase (TPO) and thyroglobulin, with TPO antibodies present in over 90% of cases. Standard treatment with thyroid hormone replacement can leave residual symptoms, highlighting the need for disease-modifying therapies. Recent advances in immunotherapy have identified Janus kinase inhibitors, such as tofacitinib and baricitinib, as promising agents for autoimmune conditions. These drugs target the Janus kinase/signal transducer and activator of transcription pathway, which mediates pro-inflammatory cytokines implicated in HT, including interferon-γ and interleukin-6. We report a case of a 44-year-old woman with both alopecia universalis and HT who experienced normalization of TPO antibody levels, no longer requiring thyroid hormone treatment following JAK inhibitor therapy for alopecia universalis—suggesting possible reversal of HT. This case highlights the immunomodulatory potential of JAK inhibitors in HT and supports further investigation into their therapeutic role in addressing both hormonal and autoimmune mechanisms in thyroid disease.

## Introduction

Hashimoto thyroiditis (HT) is the most common autoimmune disease globally, with a reported prevalence of ∼7.5% in the general population [1]. It is characterized by chronic lymphocytic inflammation of the thyroid gland, frequently leading to hypothyroidism and a range of associated metabolic dysfunctions.

Hashimoto thyroiditis is driven by a combination of cellular and humoral immune responses. Autoreactive T lymphocytes infiltrate the thyroid gland, attacking thyroid follicular cells and releasing thyroid-specific antigens, such as thyroid peroxidase (TPO) and thyroglobulin (Tg). This cellular damage initiates a humoral response, resulting in the production of autoantibodies against these antigens. TPO antibodies are present in over 90% of patients with HT. Anti-Tg antibodies are detected in 80% to 90% of cases [2].

Beyond being diagnostic markers, these autoantibodies actively contribute to thyroid tissue destruction via antibody-dependent cellular cytotoxicity and complement-dependent cytotoxicity, ultimately leading to thyrocyte apoptosis and glandular atrophy [3]. The infiltration of lymphocytes contributes to tissue inflammation by recruiting and activating neutrophils and other immune cells through their cytokine production (tumor necrosis factor-α, interferon-γ, and interleukins). The cytokines promote the inflammatory environment within the thyroid gland, leading to the recruitment and activation of additional immune cells and further perpetuating the autoimmune response [4]. As the autoimmune assault progresses, functional thyroid tissue is destroyed, resulting in hypothyroidism. High levels of TPO antibodies are directly correlated with a greater risk of developing overt hypothyroidism [5]. Common symptoms include fatigue, weight gain, cold intolerance, dry skin, constipation, and muscle weakness [6].

The standard treatment for HT-induced hypothyroidism according to the guidelines from the American Thyroid Association remains levothyroxine, a synthetic form of thyroid hormone [7]. While effective in achieving biochemical euthyroidism, a subset of patients continues to report persistent symptoms, suggesting that hormone replacement alone may not fully address the underlying autoimmune activity [8].

Recent advances in immunomodulatory therapies have introduced Janus kinase (JAK) inhibitors as promising candidates for treating autoimmune diseases. These drugs block various cytokines, including interferon-γ and γ-chain cytokines, including interleukins and tumor necrosis factor-α, which are involved in the immunopathogenesis of HT [9]. They exert their effects by inhibiting the JAK/signal transducer and activator of transcription signaling pathway, a key mechanism that transmits signals from cytokine receptors to the cell nucleus and regulates gene expression and cellular function [10, 11].

Their potential to dampen cytokine-driven inflammation suggests a novel therapeutic avenue in HT, targeting immune dysregulation at its core.

## Case presentation

A 44-year-old woman first developed alopecia areata universalis in early 2016, with complete loss of scalp and body hair over 6 weeks. Initial therapies included methotrexate, intermittent oral prednisone, topical corticosteroids, minoxidil, and cosmetic scalp prostheses.

## Diagnostic assessment

**Figure 1:**
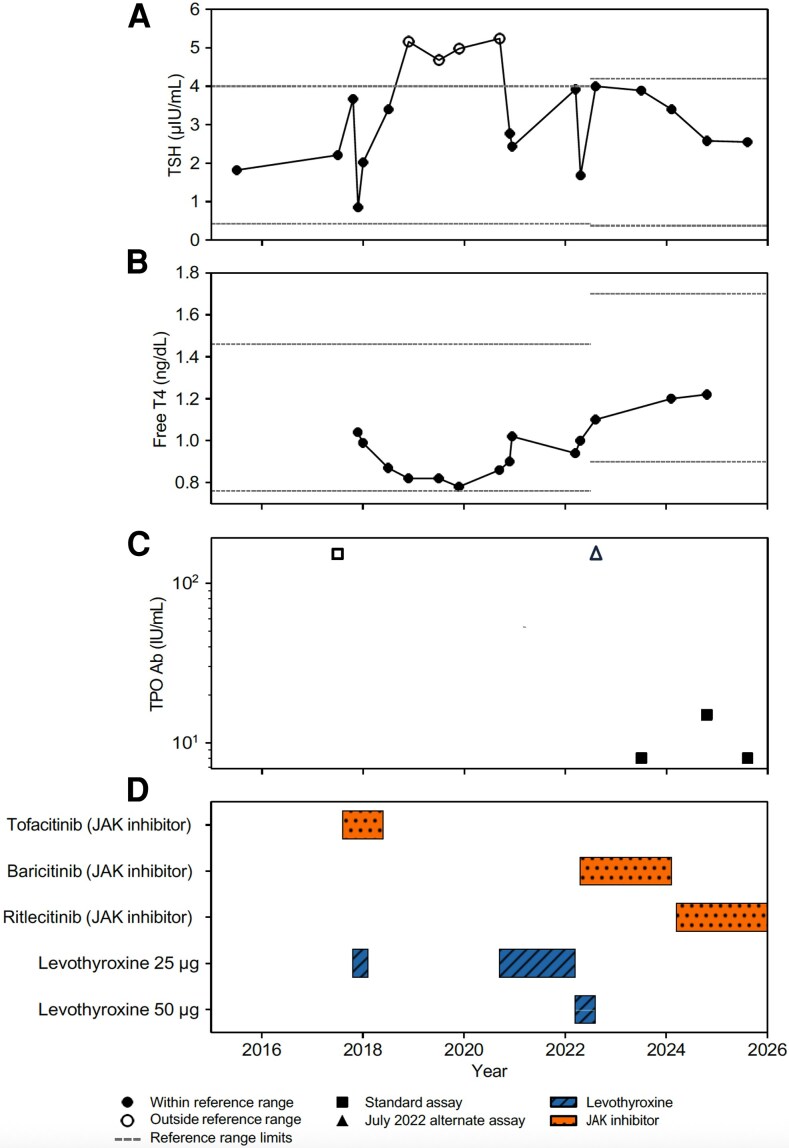
Thyroid laboratory levels and treatment history (2015-2025). Abbreviations: TSH, thyroid-stimulating hormone; free T4, free levothyroxine; TPO Ab, thyroid peroxidase antibody; JAK inhibitor, Janus kinase inhibitor. Reference ranges for thyroid function tests varied by assay and time period. TSH: 0.4 to 4.0 μIU/mL (0.4-4.5 mIU/L) from 2015 to April 2022, and 0.3 to 4.2 μIU/mL (0.3-4.2 mIU/L) from July 2022 to present. Free T4: 0.76 to 1.46 ng/dL (9.78-18.79 pmol/L) from 2015 to April 2022, and 0.9 to 1.7 ng/dL (11.58-21.88 pmol/L) from July 2022 to present. TPO Ab: <35 IU/mL. ΔThe July 2022 TPO Ab measurement was performed at Mayo Clinic using an alternate assay with a reference range <9 IU/mL. Thyroglobulin antibodies (TgAb): <40 IU/mL.

In 2017, during an evaluation for fatigue and cold intolerance, her thyroid-stimulating hormone (TSH) level was within normal limits at 2.21 µIU/mL (SI 2.21 mIU/L; reference range, 0.4-4.0 µIU/mL [SI 0.4-4.0 mIU/L]; Fig. 1A, Table 1), but TPO antibodies were elevated at 150 IU/mL (SI 150 kIU/L; reference range <35 IU/mL [SI <35 kIU/L]; Fig. 1B), measured by a chemiluminescent immunoassay (University of Minnesota). The patient did not report any neck-related symptoms. On examination, the thyroid gland was normal in size and consistency, without palpable nodules or tenderness.

**Table 1:** Thyroid laboratory values (2015-2026)

Year	Month	TSH	Free T4	TPO Ab	TgAb
2015	Jul	1.82 µIU/mL (1.82 mIU/L)	ND	ND	ND
2017	Jun	2.21 µIU/mL (2.21 mIU/L)	ND	**150 (IU/mL)**	ND
2017	Oct	3.67 µIU/mL (3.67 mIU/L)	ND	ND	ND
2017	Nov	0.85 µIU/mL (0.85 mIU/L)	1.04 ng/dL (13.39 pmol/L)	ND	ND
2018	Jan	2.02 µIU/mL (2.02 mIU/L)	0.99 ng/dL (12.74 pmol/L)	ND	ND
2018	Jun	3.40 µIU/mL (3.40 mIU/L)	0.87 ng/dL (11.2 pmol/L)	ND	ND
2018	Dec	**5.16 µIU/mL** **(5.16 mIU/L)**	**0.82 ng/dL** **(10.55 pmol/L)**	ND	ND
2019	Jun	**4.68 µIU/mL** **(4.68 mIU/L)**	**0.82 ng/dL** **(10.55 pmol/L)**	ND	ND
2019	Dec	**4.98 µIU/mL** **(4.98 mIU/L)**	**0.78 ng/dL** **(10.04 pmol/L)**	ND	ND
2020	Sep	**5.24 µIU/mL** **(5.24 mIU/L)**	**0.86 ng/dL** **(11.07 pmol/L)**	ND	ND
2020	Nov	2.77 µIU/mL (2.77 mIU/L)	0.90 ng/dL (11.58 pmol/L)	ND	ND
2020	Dec	2.43 µIU/mL (2.43 mIU/L)	1.02 ng/dL (13.13 pmol/L)	ND	ND
2022	Mar	3.92 µIU/mL (3.92 mIU/L)	0.94 ng/dL (12.1 pmol/L)	ND	ND
2022	Apr	1.68 µIU/mL (1.68 mIU/L)	1.00 ng/dL (12.87 pmol/L)	ND	ND
2022	Jul	4.00 µIU/mL (4.00 mIU/L)	1.10 ng/dL (14.16 pmol/L)	**166** *^a^* **(IU/mL)**	ND
2023	Jun	3.89 µIU/mL (3.89 mIU/L)	ND	<10 (IU/mL)	ND
2023	Jul			<10 (IU/mL)	<20 (IU/mL)
2024	Jan	3.40 µIU/mL (3.40 mIU/L)	1.20 ng/dL (15.45 pmol/L)	ND	ND
2024	Oct	2.58 µIU/mL (2.58 mIU/L)	1.22 ng/dL (15.70 pmol/L)	15 (IU/mL)	ND
2025	Jul	2.55 µIU/mL (2.55 mIU/L)	ND	<10 (IU/mL)	<20 (IU/mL)

Abnormal values are shown in bolded font. Values in parentheses are International System of Units (SI). Reference ranges for thyroid function tests varied by assay and time period. Thyroid-stimulating hormone (TSH): 0.4 to 4.0 μIU/mL (0.4-4.5 mIU/L) from 2015 to April 2022, and 0.3 to 4.2 μIU/mL (0.3-4.2 mIU/L) from July 2022 to present. Free thyroxine (FT4): 0.76 to 1.46 ng/dL (9.78-18.79 pmol/L) from 2015 to April 2022 and 0.9 to 1.7 ng/dL (11.58-21.88 pmol/L) from July 2022 to present. Thyroid peroxidase antibodies (TPO Ab): <35 IU/mL.

*^a^* The July 2022 TPO Ab measurement was performed at Mayo Clinic using an alternate assay with a reference range <9 IU/mL. Thyroglobulin antibodies (TgAb): <40 IU/mL.

## Treatment

By October 2017, her TSH had risen to 3.67 µIU/mL (SI 3.76 mIU/L) and treatment with 25 mcg levothyroxine (LT4) daily (Fig. 1A and 1C, Tables 1 and 2) was initiated as a therapeutic trial for her symptoms of fatigue and cold intolerance.

**Table 2:** Treatment exposure

Medication	Dose	Start	Stop
Tofacitinib (JAK inhibitor)	5 mg twice daily	Aug 2017	Jul 2018
Levothyroxine	25 mcg daily	Oct 2017	Jan 2018
Levothyroxine	25 mcg daily	Sep 2020	Mar 2022
Levothyroxine	50 mcg daily	Mar 2022	Jul 2022
Baricitinib (JAK inhibitor)	4 mg daily	Apr 2022	Mar 2024
Ritlecitinib (JAK inhibitor)	50 mg daily	Mar 2024	Ongoing

Abbreviation: JAK inhibitor, June kinase inhibitor.

She reported no improvement in symptoms, and levothyroxine was discontinued in January 2018. Between August 2017 and July 2018, she received tofacitinib, a JAK inhibitor, and experienced initial hair regrowth (Fig. 1C; Table 2). However, the medication was discontinued due to insurance issues.

In September 2020, she was reevaluated by endocrinology for fatigue, cold intolerance, and constipation. Laboratory testing showed an elevated TSH of 5.24 µIU/mL and a low free T4 of 0.86 ng/dL (SI 11.07 pmol/L; reference, 0.9-1.7 ng/dL [SI 11.58-21.88 pmol/L]). Levothyroxine was restarted at 25 mcg daily and subsequently increased to 50 mcg in March 2022 due to persistent hypothyroid symptoms.

In April 2022, the patient began treatment with baricitinib, another JAK inhibitor, and achieved a robust alopecia universalis response. While taking levothyroxine 50 mcg daily, the TSH remained within normal limits.

## Outcome and follow-up

At a July 2022 evaluation at Mayo Clinic, TSH was 4.0 µIU/mL (SI 4.0 mIU/L; reference, 0.3-4.2 µIU/mL SI [0.3-4.2 mIU/L]), free T4 was normal at 1.1 ng/dL (SI 14.1 pmol/L), and TPO antibodies remained elevated at 166.3 IU/mL (SI 166.3 kIU/L; reference <9 IU/mL [SI <9 kIU/L], Mayo Clinic immunoenzymatic assay). Following this assessment, the patient elected to discontinue levothyroxine, as she was uncertain whether the therapy was contributing to improvement in her symptoms of fatigue and cold intolerance.

Laboratory testing in June 2023 showed a TSH of 3.89 µIU/mL (SI 3.89 mIU/L), and notably, TPO antibodies were undetectable on University of Minnesota (UMN) chemiluminescent immunoassay (Fig. 1A and 1B, Table 1). This finding persisted in July 2023, at which time anti-Tg antibodies were also undetectable. The patient reported feeling significantly better.

The patient's alopecia initially responded to treatment with baricitinib, but efficacy gradually diminished over time. Consequently, in March 2024, treatment was transitioned to ritlecitinib tosylate.

At her most recent evaluation in July 2025, thyroid function testing revealed a TSH concentration of 2.55 µIU/m (SI 2.55 mIU/L), with both TPO and anti-Tg antibodies undetectable by UMN chemiluminescent immunoassay (Fig. 1A and 1B, Table 1). Clinically, at her last office visit from July 2025, the patient denied fatigue or other thyroid-related symptoms.

## Discussion

A substantial proportion of patients with HT continue to experience symptoms despite normalization of thyroid function tests. The data indicate that up to 15% to 20% of patients with HT report persistent symptoms despite achieving biochemical euthyroidism with levothyroxine therapy [7]. This phenomenon is attributed to factors such as ongoing thyroid autoimmunity, elevated TPO antibody titers, impaired conversion of levothyroxine to liothyronine, degrees of residual endogenous thyroid function, and nonthyroidal contributors including psychological distress and comorbidities [12].

The American Thyroid Association guidelines highlight that the presence of TPO antibodies, even in euthyroid patients, is associated with increased symptom burden and reduced quality of life, independent of thyroid hormone levels [7]. An association of higher TPO antibodies with lower quality of life and various other symptoms was shown in a group of women with euthyroid benign multinodular goiter [6]. Normalization of antithyroid antibodies in a group of women with HT undergoing total thyroidectomy was associated with resolution of severe residual hypothyroid symptoms and also indicates the role of normalization of thyroid antibodies [13].

The Whickham cohort study provides important longitudinal data on antithyroid antibody evolution in patients with autoimmune thyroid disease. Ten percent of the patients (9 females and 4 males) with initially positive thyroid antibodies did not develop evidence of thyroid dysfunction and experienced normalization of thyroid antibodies over the 20-year follow-up period. Also in this group, 10 women who had received thyroxine treatment for hypothyroidism had resolution of antithyroid antibodies [14]. The authors do suggest the possibility of false positive results, as the methodology for the detection of antithyroid antibodies in the follow-up survey had increased sensitivity and specificity compared with the techniques used in the first survey. Resolution of thyroid antibodies was also observed in a smaller study in 6 out of 38 patients with HT while on levothyroxine treatment [15]. Multiple studies have examined selenium's effect on TPO antibodies in women treated with levothyroxine. Gärtner et al reported that 9 (25%) patients in the selenium group achieved completely normalized antibody concentrations compared with 2 patients (5.8%) in the placebo group after 3 months of 200 µg daily sodium selenite supplementation [16]. In summary, spontaneous declines or normalization of thyroid antibody titers appear to be uncommon and are reported more frequently in the context of interventions, such as levothyroxine or selenium supplementation. Importantly, the existing literature does not demonstrate clear evidence of reversal of Hashimoto thyroiditis or restoration of normal thyroid function once hypothyroidism is established.

For multiple autoimmune conditions, the treatment has focused on modulating the underlying aberrant immune response. Novel treatments with JAK inhibitors have demonstrated success in autoimmune-related conditions, such as rheumatoid arthritis [17], alopecia areata [18], psoriasis [19], ulcerative colitis [20], atopic dermatitis [21], and vitiligo [22].

According to our literature review, this is the first time JAK inhibitors have been reported to normalize TPO antibodies and eliminate the need for thyroid hormone replacement, indicating a reversal of HT. Direct comparison of the effects of tofacitinib and baricitinib is not possible, as antithyroid antibody levels were not rigorously assessed during and following treatment with tofacitinib. As this patient has only mildly decreased thyroid function and elevated thyroid antibodies, it remains uncertain whether a similar response to JAK inhibitor therapy would be observed in patients with more advanced HT.

Interferon inducible chemokines play an important role in the pathogenesis of thyroid autoimmunity [23, 24]. Therefore, JAK inhibitors appear to be a promising candidate to consider as immunomodulatory treatment rather than mere thyroid hormone replacement for patients with HT with significant residual symptoms.

Although thyroid hormone replacement therapy remains the cornerstone of management for HT, it does not address the underlying autoimmune pathophysiology. While this single case report does not support the routine use of JAK inhibitors, it underscores the potential of this class of drug as a therapeutic strategy aimed at modulating the autoimmune process in Hashimoto thyroiditis. Further studies and careful assessment of the risk–benefit profile will need to be considered in a selected patient population.

## Learning points

- Symptoms of autoimmune thyroid disease may improve or resolve as antithyroid antibody levels decline or become undetectable, highlighting the correlation between immunologic activity and clinical manifestations.
- JAK inhibitors may reduce TPO antibody levels and represent a potential future therapeutic option for patients with HT who remain symptomatic despite normalization of thyroid function tests.
- JAK inhibitors may attenuate the autoimmune response in HT, similar to their effects in other autoimmune diseases, offering a potential therapeutic strategy to prevent disease progression and preserve thyroid function.

## Contributors

All authors contributed individually to authorship. A.R. was involved in the patient's diagnosis and management. A.R. was involved in the literature review and conception of the work and performed a critical review of the manuscript for important intellectual content. S.U.T. was involved in the literature review and design of the work and drafted the initial manuscript. All authors reviewed and approved the final version for publication. All authors agreed to be accountable for all aspects of the work in ensuring that any questions regarding the accuracy or integrity of any part of the manuscript are appropriately investigated and resolved.

## Data Availability

Restrictions apply to the availability of some or all data generated or analyzed during this study to preserve patient confidentiality. The corresponding author will on request detail the restrictions and any conditions under which access to some data may be provided.

## Abbreviations

HT: Hashimoto thyroiditis
JAK: Janus kinase
Tg: thyroglobulin
TPO: thyroid peroxidase
TSH: thyroid-stimulating hormone
